# Status of Insecticide Susceptibility in *Anopheles gambiae* Sensu Lato and *Anopheles funestus* Mosquitoes from Western Kenya

**DOI:** 10.1673/031.008.1101

**Published:** 2008-02-19

**Authors:** Luna Kamau, Derek Agai, Damaris Matoke, Lucy Wachira, Geoffrey Gikandi, John M. Vulule

**Affiliations:** ^1^ Centre for Biotechnology Research and Development, Kenya Medical Research Institute, P.O. Box 54840, Nairobi-00200, Kenya.; ^2^ University of Nairobi, Department of Zoology, P.O. Box 30197 Nairobi-00100, Kenya; ^3^ Centre for Vector Biology and Control Research, Kenya Medical Research Institute, P.O. Box 1578, Kisumu-40100, Kenya

**Keywords:** diagnostic bioassays, knockdown rates, percentage mortality, knockdown resistance gene

## Abstract

The status of resistance was investigated in *Anopheles gambiae* sensu lato and *An. funestus* (Diptera: Culicidae) mosquitoes from western Kenya to four classes of insecticides approved by World Health Organization for indoor residual spraying. The prevalence of the knockdown-resistance (kdr) mutation associated with resistance to pyrethroids and DDT was determined in *An. gambiae* s.l.. Standard World Health Organization diagnostic bioassay kits for DDT (an organochlorine), fenitrothion (an organophosphate), bendiocarb (a carbamate), and the pyrethoirds, lambdacyhalothrin and permethrin, were used. Knockdown every 10 min and mortality 24 h after exposure were noted. Controls not treated with insecticides and with the susceptible *An. gambiae* KISUMU strain were included in the bioassays. The presence of the kdr gene was determined using a standard diagnostic polymerase chain reaction assay. Over 98% mortality was observed for tests with all insecticides for both *An. gambiae* s.l. and *An. funestus.* Knockdown rates were not significantly different between *An. gambiae* s.l. and the KISUMU strain control. 50% and 95% knockdown times were either slightly lower than those for the KISUMU strain or higher by factors of less than 1.6. The mean frequency of the East African kdr mutation was 24.7% in *An. gambiae* sensu strictu. Based on conventional criteria where susceptibility is defined by mortality rates >98% 24 h after exposure, no evidence for resistance was found, implying that vector control measures employing any of the insecticides tested would be unhampered by resistance. The observed frequencies of the kdr mutation do not appear to compromise the effectiveness of the insecticides. The need for continuous monitoring of the status of insecticide resistance and of the impact of any observed resistance on the efficacy of vector control programs employing insecticides is apparent.

## Introduction

*Anopheles gambiae* sensu stricto, *An. arabiensis* and *An. funestus* are the most important vectors of malaria in sub-Saharan African. Vector control strategies such as the use of insecticides both for indoor residual spraying and for the treatment of bed nets have been shown to have significant impact on the transmission of the malaria (Hawley et al. 2003; Mabaso et al. 2004). There is, however, an accumulation of evidence of resistance by the malaria vectors to commonly used insecticides (WHO 1992). Among the different mechanisms of insecticide resistance, the knockdown resistance (kdr) mechanism, which results from mutations in the voltage-gated sodium channel (the target-site for DDT and pyrethroids), and metabolic resistance, which occurs when levels of insecticide-detoxifying enzymes are elevated or their activity modified, are the most important (Brogdon and McAllister 1998). In Kenya, the first reported case of resistance was in the context of insecticide-treated net use in Western Kenya where reduced knockdown rates were seen (Vulule et al. 1994). Stump et al. (2004) also found significant differences in kdr gene frequency between the large-scale insecticide treated net trial Asembo area and other areas in Western Kenya. More recently, studies in Central Kenya found no evidence for insecticide resistance in *An. arabiensis* (Kamau et al. 2006). Reports of insecticide resistance are more abundant in West Africa where different levels of resistance have been found even within short distances and during different seasons (Diabate et al. 2002, 2004; Awolola et al. 2003; Yawson et al. 2004). Other insecticide resistance studies involving *An. albimanus* suggest that levels of resistance can increase significantly within short periods of time, such as within six months, and that the dominant resistance mechanism may be highly localized (Brogdon et al. 1988a, 1988b).

The current study is the first report of the status of phenotypic resistance to insecticides in *An. funestus* in Kenya and in *An. gambiae* outside the area of intensive insecticide treated net use. Although the aforementioned studies indicate that resistance levels are generally low, sustained use of insecticides may result in increased resistance that would threaten the sustainability of this vector control strategy. Thus, continued monitoring of resistance is necessary. The status of resistance in *An. funestus* and *An. gambiae* s.l. to insecticides was determined in each of the four classes of insecticides that have been approved for indoor residual spraying by WHO, namely DDT (an organochlorine), fenitrothion (an organophosphate), bendiocarb (a carbamate), the pyrethroids, lambdacyhalothrin and permethrin, as well as the frequency of the kdr gene in *An. gambiae* s.l.

## Materials and Methods

### Study sites

Mosquito specimens were collected from Ahero and Rota in Western Kenya. Ahero (0° 10′ S, 34° 55′ E) is a rice irrigation area where large-scale rice farming was carried out prior to the 1990s. Total acreage under rice irrigation was reduced drastically in the years that followed due to reduced governmental support for the management of water distribution and purchase of produce and has just started increasing with renewed support. Carbofuran, applied in the seed furrows, was used to control pests (Wandiga et al. 2003). Rota (0° 08′ S, 34° 36′ E), is on the shores of Lake Victoria. Residents are largely fishermen although subsistence farming is practiced with very little, or no, use of chemical fertilizers and pesticides. Various scientists from the Kenya Medical Research Institute (KEMRI) have conducted studies in both Ahero and Rota since the 1930s and the working relations between KEMRI staff and the local residents are cordial. Additionally, specimens from Kisii in Western Kenya (0° 46′ S, 34° 56′ E), Mwea in Central Kenya (0° 44′ S, 36° 79′ E) and Kwale at the Kenyan Coast (4° 11′ S, 39° 26′ E), collected between 2003 –2005, were also analyzed for the presence the kdr mutation.

### Specimen collection, identification and rearing

*An. gambiae* s.l. mosquitoes were sampled from Rota. Two samples were taken, one during the short rains between 12^th^–16^th^ September 2005 and the other during the long rains between 24^th^–28^th^ April 2006. For *An. funestus*, two dry season samples were taken from Ahero (7^th^–11^th^ February 2005 and 14^th^–18^th^ March 2005) and one during the long rains from 29^th^–May-3^rd^ June 2005. Additional *An. funestus* samples were taken from Rota between 4^th^–8^th^ July 2005 and 13^th^–16^th^ September 2005 during the long and short rains, respectively. Adult female mosquitoes were collected from walls inside human dwellings by manual aspiration and identified as *An. gambiae s.l.* and *An. fanestus* based on morphological characteristics (Gillies and De Meillon 1968). Individual mosquitoes were then allowed to oviposit and Fi families were raised separately. *Anopheles gambiae* s.l. mosquitoes were further identified to sibling species of the *An. gambiae* complex after oviposition using species-specific polymerase chain reaction technique of Scott et al. (1993) following DNA extraction by the alcohol precipitation method of Collins et al. (1987). Over 400 gravid or half gravid *An. gambiae* s.l. specimens and a similar number of *An. funestus* were collected during the entire sampling period but the number of families included in the bioassay were much lower (Table 1) because some mosquitoes died before ovipositing, others failed to oviposit and some family sizes were very low.

### Insecticide susceptibility bioassays

Insecticide susceptibility assays were performed on adult non-blood fed mosquitoes 1–3 days old using WHO mosquito diagnostic test kits as previously described (Kamau et al. 2006). Only one specimen from each isofemale line was used in each of the bioassays except where bioassays were conducted to test for variability in responses between male and female mosquitoes. For this test, one male and one female mosquito from a particular isofemale line were used in the assay. Susceptibility was tested for DDT, fenitrothion, bendiocarb, lambdacyhalothrin and permethrin. Insecticide knockdown effects were recorded every 10 min and mortality 24 h after exposure noted. Control tests in which bioassays were conducted using papers treated only with silicone oil were also included. An additional control that was included in the bioassay was *An. gambiae* s.l. in which the *An. gambiae* KISUMU susceptible strain provided by Dr. J. Vulule was used.

**Table 1.**
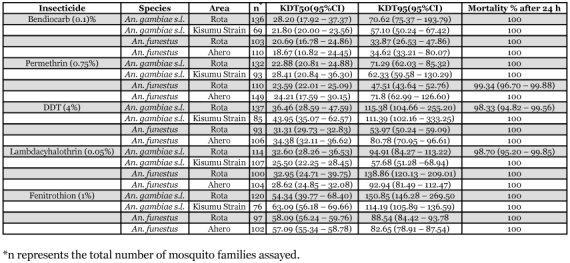
Insecticide resistance bioassays using diagnostic doses of each of the insecticides. The Table shows KDT_50_ and KDT_95_ values and percentage mortality 24h post-exposure for the *An. gambiae s.l.* and *An. funestus* samples tested.

### Knockdown resistance (kdr) gene analysis

The presence of the knockdown resistance (kdr) was tested using standard diagnostic PCR assays. Both the L1014S (leucine-serine) kdr allele found in East Africa (Ranson et al 2000) and the L1014F (leucine-phenylalanine) kdr allele found in West Africa (Martinez-Torres 1998) were assayed for using field-collected specimens. DNA from a proportion of specimens was also directly sequenced in a double-blind assay to re-confirm the presence of the kdr mutation. Sequencing was performed using the ABI 3700 sequencer following DNA amplification and purification using a Qiagen purification kit at CDC, Atlanta Georgia.

### Statistical analyses

The WHO (1992) criterion for evaluating resistance or susceptibility in mosquito populations was used in which mortality rates of less than 80% indicate resistance while those greater than 98% indicate susceptibility. Mortality rates between 80–98% suggest the possibility of resistance that needs to be clarified. Analysis of variance (ANOVA) was used to compare knockdown rates between different samples. Fifty and 95% knockdown times (KDT50 and KDT95) were estimated by the log-time probit model using the *LdP Line*^R^ software (Ehabsoft).

## Results

### Insecticide susceptibility in *An. gambiae *s.l. mosquitoes

The ratio of *An. arabiensis* to *An. gambiae* s.s among mosquitoes included in the insecticide resistance bioassays ranged from 1:2.2 – 1:3 for the different insecticides. To determine whether responses to exposure to the different insecticides were affected by sex, separate bioassays were run for male and female *An. gambiae s.l.* mosquitoes. The proportion of mosquitoes knocked down after insecticide exposure was not significantly different between the two sexes for all five insecticides tested (ANOVA, *P* >0.100 for all tests). Subsequent bioassays were run without separating female and male mosquitoes but keeping their proportions approximately balanced.

All mosquitoes were knocked down after exposure with insecticides for all insecticides except DDT. However, knockdown rates with DDT were not significantly different between the samples taken in 2005 and those taken in 2006 (ANOVA, F = 0.281, df = 1,8, *P* = 0.610) with the mean percentage of mosquitoes knocked down after exposure being 93.4 (95% CI 87.9–96.9). Mortality rates 24 h after exposure were 100% with permethrin, bendiocarb and fenitrothion but were slightly reduced with lambdacyhalothrin and DDT (Table 1). Mortality 24 h after exposure was 100% for the *An. gambiae* susceptible Kisumu Strain control for all the insecticides tested. Fifty and 95% knockdown times (KDT_50_ and KDT_95_) for the field samples compared well with those of the Kisumu strain control and differed by factors of between 0.6 and 1.6 (Table 1), suggesting that the test specimens are best categorized as susceptible.

### Insecticide susceptibility in *An. funestus *mosquitoes

All *An. funestus* mosquitoes were knocked down after exposure with bendiocarb and fenitrothion but not with permethrin, DDT and lambdacyhalothrin. Differences in knockdown rates with these three insecticides were however not significantly different between the various samples taken (ANOVA, *P* >0.100 for all tests).

**Table 2.**
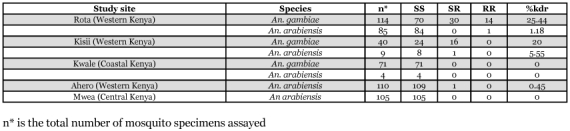
The distribution of Leucine-Serine (East African) kdr mutation among *An. gambiae* s.l. populations from Kenya.

Knockdown rates at the end of exposure were however much reduced for exposure to lambdacyhalothrin for mosquitoes collected in Ahero (86.5%, 95% CI 784–92.4%) and Rota (89.0%, 95% CI 81.2–94.4%) and also for exposure to permethrin and DDT in Ahero which were 86.6% (95% CI 80.0–91.6%) and 84.0% (95% CI 75.6–90.4%), respectively. Fifty and 95% knockdown times (KDT_50_ and KDT_95_) are provided in Table 1. Mortality 24 h after exposure was 100% for all insecticides tested for the Rota collections and for all the Ahero collection except with permethrin (mean mortality 99.0%, 95% CI 95.0–99.9%).

Within-family variation in resistance was tested using a total of 158 *An. funestus* mosquitoes belonging to 8 isofemale lines (mean family size 19.75 ± 1.82, range 12–25). Mortality 24 h after exposure with permethrin was 100% for all the families, with mean knockdown rate at the end of the exposure with the insecticide standing at 98.7% (95% CI 95.5 – 99.1%)

### Prevalence of the knockdown resistance (kdr) gene *in An. gambiae* s.l. mosquitoes

A total of 27 specimens (10 homozygous for the L1014S mutation, 7 homozygous for the wild type susceptible allele and 10 heterozygotes) as scored by the PCR assay were sequenced. The sequence data matched the PCR data perfectly, re-confirming the reliability of results obtained by the allele-specific PCR. Table 2 summarizes the results of the leucine-serine (East African) kdr mutation among the *An. gambiae* s.l. populations studied.

The leucine-phenylalanine (West African) mutation was absent in all 674 alleles that were sampled (Rota, n = 238; Kisii, n = 80; Kwale = 100; Mwea = 154; Ahero = 102).

## Discussion

The status of resistance to DDT, fenitrothion, bendiocarb, lambdacyhalothrin and permethrin was investigated in *An. gambiae* s.l. and *An. funestus* mosquitoes from Western Kenya. Based on the conventional criteria for characterizing insecticide resistance/susceptibility, where susceptibility is defined by mortality rates greater than 98% 24 h after exposure, no evidence for resistance was found. In addition, KDT50 and KDT95 obtained in the present study are similar to those observed for *An. gambiae* s.l. populations that are categorized as susceptible by different investigators (Yawson et al. 2004; Kristan et al. 2003; Chandre et al. 1999). Thus our results imply that the use of any of these insecticides in vector control programs would be unhampered by resistance.

However, considerable levels of the kdr gene, which has been associated with resistance to pyrethroids and DDT among *An. gambiae* mosquitoes were found, indicating developing resistance. The low levels of the kdr gene in *An. arabiensis* that were observed are similar to those previously observed in other regions including an area where insecticide treated nets have been used for a long time in Western Kenya (Stump et al. 2004) and in West Africa (e.g. Diabate et al. 2002; Kristan et al. 2003; Fanello et al. 2003). None of the specimens that were assayed for the leucine - phenylalanine kdr mutation that is common in West Africa were positive. However, recent studies in the East African region have recorded the presence of this mutation in both *An. gambiae* and *An. arabiensis* though at very low frequencies (Kulkarni et al. 2006; Verhaeghen et al. 2006). This suggests that the geographical range of this mutation extends beyond Western Africa and that both kdr mutations should be tested in studies of insecticide resistance. The significant presence of the kdr mutation in *An. gambiae* mosquitoes in the present study may originate either from selection pressure exerted by insecticide use or from migration of resistant mosquitoes into the study areas. The use of insecticides for malaria control and of pesticides in agriculture is however generally low in Rota and Kisii. Because the area associated with a deme in *An. gambiae* has been found to be quite large (e.g. Lehmann et al. 1996; Kamau et al. 1998; Onyabe and Conn 2001), migration of resistant mosquitoes from areas experiencing high selection intensities cannot be ruled out.

Brogdon and McAllister (1998) have argued that insecticide resistance should be a concern only if it compromises the efficacy of intervention programs employing the particular insecticides. It is however an open question as to what level of resistance would compromise vector control programs. This is because studies conducted in different areas have yielded somewhat discordant results. In the study reported here for example, the presence of relatively high levels of the kdr gene was apparently not correlated with phenotypic resistance. These findings are in concordance with those obtained in West Africa where various researchers have found insecticides to be highly effective even in the face of kdr prevalence of up to 94% among mosquito populations (e.g. Chandre et al. 2000; Kristan et al. 2003; Henry et al. 2005). Similarly, studies in India found indoor spraying with DDT to be effective against *An. culicifacies* populations that revealed only 21.4% mortality in resistance bioassays with DDT (Sharma et al 2005). On the other hand, in a recent study in experimental huts in Benin, N'guessan and others (2007) found mosquito feeding to be inhibited and mortality to be only 30% in an *An. gambiae* population with a kdr frequency of 83%, providing strong evidence that resistance may interfere with the effectiveness of vector control measures. Chandre et al. (2000) have suggested that the apparently paradoxical phenomenon whereby insecticide resistance does not undermine control measures may be due to reduced irritant effects associated with resistance that allow resistant mosquitoes to stay longer on insecticide treated surfaces and thus acquire lethal doses of the insecticide.

Thus, there is need not only for continuous monitoring of the status of insecticide resistance in different settings, but also for the assessment of the impact of any observed resistance on the effectiveness of vector control programs. The results of this study provide baseline information essential in the monitoring of the development of insecticide resistance in Kenya. Knowledge of resistance mechanisms will enable informed selection of alternative insecticides for vector control programs in the face of resistance.

## Abbreviations

kdr: knockdown resistance;
KDT_50_, KDT_95_: 50, 95% knockdown times;
WHO: World Health Organization

## References

[bibr01] Awolola TS, Brooke BD, Koekemoer LL, Coetzee M (2003). Absence of the *kdr* mutation in the molecular ‘M’ form suggests different pyrethroid resistance mechanisms in the malaria vector mosquito *Anopheles gambiae* s.s.. *Tropical Medicine and International Health*.

[bibr02] Brogdon JH, Hobbs WG, Jean Y, Jacques JR, Charles LB (1988a). Microplate assay analysis of reduced fenitrothion susceptibility in Haitian *Anopheles albimanus*.. *Journal of the American Mosquito Control Association*.

[bibr03] Brogdon WG, Beach RF, Stewart JM, Castanaza L (1988b). Microplate assay analysis of the distribution of organophosphate and carbamate resistance in Guatemalan *Anopheles albimanus*.. *Bulletin of the World Health Organization*.

[bibr04] Brogdon WG, McAllister JC, Insecticide resistance and vector control. (1998). *Emerging Infectious Diseases*.

[bibr05] Chandre F, Manguin S, Brengues J, Dossou YJ, Darriet F, Diabate A, Carnevale P, Guillet P (1999). Current distribution of pyrethroid resistance gene (kdr) in *Anopheles gambiae* complex from West Africa and further evidence for reproductive isolation of Mopti form.. *Parasitologia*.

[bibr06] Chandre F, Darriet F, Duchon S, Finot L, Manguin S, Carnevale P, Guillet P (2000). Modifications of pyrethroid effects associated with kdr mutation in *An. gambiae*.. *Medical and Veterinary Entomology*.

[bibr07] Collins FH, Mendez MA, Rasmussen MO, Mehaffey PC, Besansky NJ, Finnerty V (1987). A ribosomal RNA gene probe differentiates member species of the *Anopheles gambiae* complex.. *American Journal of Tropical Medicine and Hygiene*.

[bibr08] Diabate A, Baldet T, Chandre F, Akogbeto M, Guiguemde TR, Darriet F, Brengues C, Guillet P, Hemingway J, Small GH, Hougard JM (2002). The role of agricultural use of insecticides in resistance to pyrethroids in *Anopheles gambiae s.l.* in Burkina Faso.. *American Journal of Tropical Medicine and Hygiene*.

[bibr09] Diabate A, Brengues C, Baldet T, Dabire KR, Hougard JM, Akogbeto M, Kengne P, Simard F, Guillet P, Hemingway J, Chandre F (2004). The spread of the Leu-Phe kdr mutation through *Anopheles gambiae* complex in Burkina Faso: genetic introgression and de novo phenomena.. *Tropical Medicine and International Health*.

[bibr10] Fanello C, Petrarca V, Della Torre A, Santolamazza F, Dolos G, Coulibaly M, Alloueche A, Curtis CF, Toure YT, Coluzzi M (2003). The pyrethroid knockdown resistance gene in the *Anopheles gambiae* complex in Mali and further indication of incipient speciation within *An. gambiae* s.s.. *Insect Molecular Biology*.

[bibr11] Gillies MT, DeMeillon B (1968). The Anophelinae of Africa South of the Sahara (Ethiopian Zoogeographical Region).. *Publications of the South African Institute for Medical Research*.

[bibr12] Hawley WA, Phillips-Howard PA, ter Kuile FO, Terlouw DJ, Vulule JM, Ombok M, Nahlen BL, Gimnig JE, Kariuki SK, Kolczak MS, Hightower AW (2003). Community-wide effects of permethrin-treated bed nets on child mortality and malaria morbidity in western Kenya.. *American Journal of Tropical Medicine and Hygiene*.

[bibr13] Henry MC, Assi SB, Rogier C, Dossou-Yovo J, Chandre F, Guillet P, Carnevale P (2005). Protective efficacy of lambda-cyhalothrin treated nets in *Anopheles gambiae* pyrethroid resistance areas of Cote d'Ivoire.. *American Journal of Tropical Medicine and Hygiene*.

[bibr14] Kamau L, Lehmann T, Hawley WA, Orago ASS, Collins FH (1998). Microgeographic genetic differentiation of *Anopheles gambiae* mosquitoes from Asembo Bay, Western Kenya: A comparison with Kilifi in coastal Kenya.. *American Journal of Tropical Medicine and Hygiene*.

[bibr15] Kamau L, Vulule JM (2006). Status of Insecticide susceptibility in *Anopheles arabiensis* from Mwea rice irrigation scheme, Central Kenya.. *Malaria Journal*.

[bibr16] Kristan M, Fleischmann H, Della Torre A, Stich A, Curtis CF (2003). Pyrethroid resistance/susceptibility and differential Urban/rural distribution of *Anopheles arabiensis* and *An. gambiae* s.s. malaria vectors in Nigeria and Ghana.. *Medical and Veterinary Entomology*.

[bibr17] Kulkarni MA, Rowland M, Alifrangis M, Mosha FW, Matowo J, Malima R, Peter J, Kweka E, Lyimo I, Magesa S, Salanti A, Rau ME, Drakeley C (2006). Occurrence of the leucine-to-phenylalanine knockdown resistance (kdr) mutation in *Anopheles arabiensis* populations in Tanzania, detected by a simplified high-throughput SSOP-ELISA method.. *Malaria Journal*.

[bibr18] Lehmann T, Besansky NJ, Hawley WA, Fahey TG, Kamau L, Collins FH (1996). Microgeographic structure of *Anopheles gambiae* in Western Kenya based on mtDNA and microsatellite loci.. *Molecular Entomology*.

[bibr19] Mabaso ML, Sharp B, Lengeler C (2004). Historical review of malarial control in southern African with emphasis on the use of indoor residual house-spraying.. *Tropical Medicine and International Health*.

[bibr20] Martinez-Torres D, Chandre F, Williamson MS, Darriet F, Berge JB, Devonshire AL, Guillet P, Pasteur N, Pauron D (1998). Molecular characterization of pyrethroid knockdown resistance (kdr) in the major Malaria vector *Anopheles gambiae* s.s.. *Insect Molecular Biology*.

[bibr21] N'Guessan R, Corbel V, Akogbeto M, Rowland M (2007). Reduced efficacy of insecticide-treated nets and indoor residual spraying for malaria control in pyrethroid resistance area, Benin.. *Emerging Infectious Diseases*.

[bibr22] Onyabe DY, Conn JE (2001). Genetic differentiation of the malaria vector *Anopheles gambiae* across Nigeria suggests that selection limits gene flow.. *Heredity*.

[bibr23] Ranson H, Jensen B, Vulule JM, Wang X, Hemingway J, Collins FH (2000). Identification of a point mutation in the voltage-gated sodium channel gene of Kenyan *Anopheles gambiae* associated with resistance to DDT and pyrethroids.. *Insect Molecular Biology*.

[bibr24] Scott JA, Brogdon WG, Collins FH (1993). Identification of single specimens of the *Anopheles gambiae* complex by PCR reaction.. *American Journal of Tropical Medicine and Hygiene*.

[bibr25] Sharma SN, Shukla RP, Raghavendra K, Subbarao SK (2005). Impact of DDT spraying on malaria transmission in Bareilly District, Uttar Pradesh, India.. *Journal of Vector Borne Diseases*.

[bibr26] Stump AD, Atieli FK, Vulule JM, Besansky NJ (2004). Dynamics of the pyrethroid knockdown resistance allele in Western Kenya populations of *Anopheles gambiae* in response to Insecticide-treated bed net trial.. *American Journal of Tropical Medicine and Hygiene*.

[bibr27] Verhaeghen K, Van Bortel W, Roelants P, Backeljau T, Coosemans M (2006). Detection of the East and West African kdr mutation in *Anopheles gambiae* and *Anopheles arabiensis* from Uganda using a new assay based on FRET/Melt Curve analysis.. *Malaria Journal*.

[bibr28] Vulule JM, Beach RF, Atieli FK, Roberts JM, Mount DL, Mwangi RW (1994). Reduced susceptibility *of An. gambiae* to permethrin associated with the use of permethrin-impregnated bed nets and curtains in Kenya.. *Medical and Veterinary Entomology*.

[bibr29] Wandiga SO, Lalah JO, Kaigwara PN, Taylor DM, Klaine SJ, Carvalho FP, Barcel D, Everaart J (2003). Pesticides in Kenya.. *Pesticides in Coastal Tropical Ecosystems: Distribution, Fate and Effects.*.

[bibr30] WHO (1992). Vector resistance to insecticides. 15^th^ Report of the WHO Expert Committee on Vector Biology and Control.. *World Health Organization Technical Report Series*.

[bibr31] Yawson AE, McCall PJ, Wilson MD, Donnelly MJ (2004). Species abundance and insecticide resistance of *Anopheles gambiae* in selected areas of Ghana and Burkina Faso.. *Medical and Veterinary Entomology*.

